# Species‐specific drivers explain fish feeding and individual niche variation

**DOI:** 10.1111/jfb.70136

**Published:** 2025-07-13

**Authors:** Javier Sánchez‐Hernández, Iñaki Fernández de Larrea, Ioar de Guzmán, José M. González, Aitor Larrañaga

**Affiliations:** ^1^ Área de Biodiversidad y Conservación, Departamento de Biología y Geología, Física y Química Inorgánica Universidad Rey Juan Carlos (URJC) Madrid Spain; ^2^ Instituto de Investigación en Cambio Global (IICG) Universidad Rey Juan Carlos, Móstoles Madrid Spain; ^3^ Department of Plant Biology and Ecology Faculty of Science and Technology, University of the Basque Country (UPV/EHU) Bilbao Spain; ^4^ Ecologie Comportementale et Biologie des Populations de Poissons (UMR Ecobiop) French National Research Institute for Agriculture, Food and Environment (INRAE) Saint‐Pée sur Nivelle France

**Keywords:** diet, fish, niche variation, riverine‐dwelling populations

## Abstract

The trophic ecology of animals often results from complex interactions between environmental and biological drivers. Yet, studies that explore trophic ecology across multiple fish species while considering a wide range of environmental variables remain limited. In this study, we analysed diet and niche variation in fish communities across five protected areas in the northern Iberian Peninsula, examining their responses along environmental and biological gradients. Our multiple regression analyses showed that both environmental and biological drivers significantly influence fish feeding behaviour and individual specialisation, but these effects are species‐specific. We propose that the primary mechanisms shaping fish feeding strategies, whether benthic foraging or surface‐drift feeding, are determined by intrinsic factors such as species‐specific feeding behaviours and ontogenetic stage, along with benthic macroinvertebrate density and fish density. Specifically, benthic macroinvertebrate density positively affected individual niche specialisation in brown trout (*Salmo trutta*) and Pyrenean stone loach (*Barbatula quignardi*), whereas it negatively impacted the Pyrenean minnow (*Phoxinus bigerri*). An increase in total fish density, measured as the number of fish per square meter, negatively influenced individual niche specialisation of brown trout. However, brown trout density had a positive effect on individual niche specialisation of Pyrenean stone loach and Pyrenean minnow. The findings have direct implications for conservation and restoration efforts, highlighting the importance of increasing habitat heterogeneity to meet species‐specific habitat needs and promoting benthic macroinvertebrate production to support fish feeding requirements.

## 1 INTRODUCTION

Trophic ecology of animals is well documented because it provides a comprehensive understanding of ecological interactions and processes (Hanley & La Pierre, 2015), although there is limited information on the mechanisms driving spatial variations in the feeding behaviour (e.g., Lunghi et al., 2020; Sánchez‐Hernández et al., 2021a). The current literature indicates that feeding is a highly flexible functional trait, showing large spatiotemporal variation (Bolnick et al., 2003; Jaeger et al., 2013; Lunghi et al., 2020; Sánchez‐Hernández, 2024). Variation in feeding and predator niches has been associated with several drivers such as competitive interactions among sympatric species (Araújo et al., 2011; Costa‐Pereira et al., 2018), which can be framed within the niche variation hypothesis suggesting that individuals within populations tend to display niche expansion when they are released from interspecific competition (Van Valen, 1965). Other drivers of predator niche variation include population density as a proxy of intraspecific competition (e.g., Araújo et al., 2011; Svanbäck & Bolnick, 2007; Tinker et al., 2012; Ward et al., 2006), prey availability (e.g., Tinker et al., 2008; Araújo et al., 2011; Sánchez‐Hernández et al., 2021a), predation risk (Araújo et al., 2011) and morphological constrains linked to body size (Sánchez‐Hernández et al., 2021a). Thus, feeding patterns are often a result of complex interactions between environmental and biological drivers (e.g., Clavero et al., 2003; de Camargo et al., 2014; Sánchez‐Hernández, Finstad, et al., 2019).

Bottom‐up mechanisms have been commonly considered by researchers as an important mechanism explaining trophic niche and feeding patterns of fish species (Sjödin et al., 2018; Sánchez‐Hernández et al., 2021a). Most fish species exhibit flexible feeding behaviours, adjusting their diets to take advantage of pulsed resources, such as the high availability of terrestrial arthropods during summer, when the aquatic invertebrate biomass is usually low, or the emergence of aquatic insects peaking around spring and early summer in temperate systems when the peak of terrestrial invertebrates has not occurred yet (Nakano & Murakami, 2001; Sweeney & Vannote, 1982). The ability to forage at different depths in the water column has been recognised as key to understand differences in feeding (bottom‐autochthonous vs. surface‐allochthonous feeding) at individual and population levels and coexistence of sympatric species in stream‐dwelling fish communities through food resource partitioning (Horká et al., 2017; Nakano et al., 1999; Sánchez‐Hernández et al., 2016). For example, when benthic macroinvertebrates are more abundant and accessible than surface prey, riverine brown trout (*Salmo trutta*) is unlikely to specialise in surface‐drift foraging (Sánchez‐Hernández & Cobo, 2018a). Many researchers have also provided evidence of the importance of morphological constraints on feeding through ontogeny to understand feeding of fish species (Anaya‐Rojas et al., 2023; Sánchez‐Hernández, Nunn, et al., 2019). In this regard, the most common ontogenetic dietary shifts in apex fish species, such as salmonids, involve an increased consumption of allochthonous resources (terrestrial invertebrates) and fish prey (Sánchez‐Hernández, 2024 and references therein). In contrast, riverine populations of omnivorous cyprinid species, such as the northern Iberian chub (*Squalius carolitertii*), show a decrease in detritus consumption over the course of their ontogeny (Sánchez‐Hernández & Cobo, 2012).

Additionally, physical habitat characteristics (e.g., flow velocity combined with habitat complexity) can affect feeding conditions, as fish individuals commonly choose feeding patches with current velocities that allow them to maintain their position and feed effectively (Orlov et al., 2006). For example, higher substratum roughness reduces the conspicuousness and accessibility of benthic invertebrates to brown trout (De Crespin Billy & Usseglio‐Polatera, 2002). Understanding the drivers of fish feeding allows ecologists to better predict how environmental changes, such as climate change, pollution and habitat destruction, will impact both their population and food webs and may be useful for fishery management practices. However, research on this topic is typically biased towards common model organisms and socio‐economically significant stream‐dwelling species, such as salmonids (e.g., Evangelista et al., 2014; Orlov et al., 2006; Sánchez‐Hernández & Cobo, 2018b), as well as other fish species of socio‐economic importance inhabiting lacustrine and marine environments (e.g., Hayden et al., 2019; Sánchez‐Hernández et al., 2021b). To identify general patterns in the drivers of fish feeding, it is essential to expand studies to include a broader range of species, particularly omnivorous and endemic species, beyond just predatory fish.

In the present study, the primary objective was to identify the main environmental and biological drivers of feeding and individual niche variation in a diverse stream‐dwelling fish community in northern Iberian Peninsula, including both omnivorous species such as Pyrenean minnow (*Phoxinus bigerri*) and predator species such as brown trout and Pyrenean stone loach (*Barbatula quignardi*). With this, the current study represents an innovative attempt to identify the relative importance of multiple environmental and ecological factors in shaping dietary patterns, population niche width and individual specialisation across fish taxa based on the following hypotheses:

Hypothesis 1 Brown trout heavily relies on terrestrial invertebrates (Sánchez‐Hernández & Cobo, 2018a, 2018b), whereas Pyrenean stone loach primarily forages on aquatic invertebrates (Oscoz et al., 2004), and Pyrenean minnow has omnivorous behaviour, consuming both animal and non‐animal resources (Oscoz et al., 2001, 2008). These differences in diet composition are likely to be accompanied by variations in dietary specialisation at both individual and population levels, with omnivorous species expected to exhibit broader population‐level niche widths.

Hypothesis 2 Feeding differences among fish species are driven by species‐specific environmental and biological drivers. Overall, low environmental habitat complexity and high availability of aquatic prey drive species towards benthic or aquatic feeding (De Crespin Billy & Usseglio‐Polatera, 2002; Orlov et al., 2006; Sánchez‐Hernández & Cobo, 2018a). However, because fish species differ in body length, feeding strategies (predatory vs. omnivore) and habitat preferences (e.g., water column use) (e.g., Prenda et al., 1997; Doadrio, 2001), environmental and biological drivers may not affect all species equally. For example, fish length, as a proxy of ontogenetic mechanisms, may have a stronger impact on salmonids because they achieve larger maximum body length allowing them to increase both drift foraging ability and surface feeding over ontogeny (Sánchez‐Hernández, Nunn, et al., 2019).

Hypothesis 3 Differences in individual niche variation may vary among fish species. High fish densities as a proxy of inter‐ and intraspecific competition would lead weaker competitors to specialise on suboptimal resources and chiefly promote niche variation among individuals within a population, but the effect of intra‐ and interspecific competition on niche variation can be variable (see Araújo et al., 2011; Sánchez‐Hernández et al., 2021a). In this sense, fish density is likely to be particularly relevant for salmonid species, as they typically exhibit density‐dependent effects (Grossman & Simon, 2020).

## 2 METHODS

### 2.1 Study area

**FIGURE 1:**
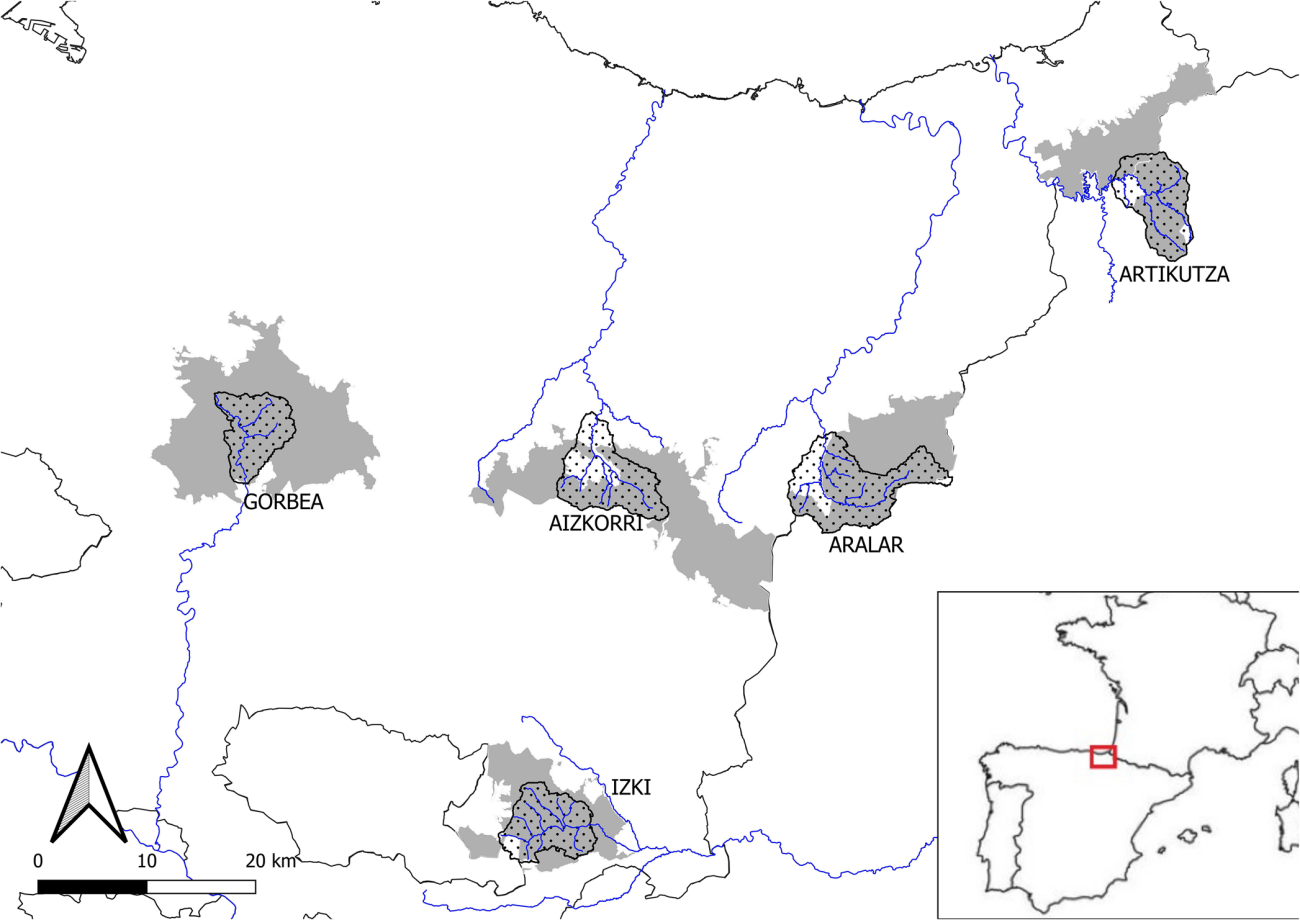
Map of the study area showing sampling locations in the five protected areas. Dotted and grey areas are the catchments of riverine systems and the protected areas, respectively.

We selected five riverine systems located within five protected areas of the Basque Country and Navarre in northern Iberian Peninsula (Figure 1). Three of these catchments (Artikutza, Izki and Gorbea) are almost entirely protected; thus they only suffer from mild anthropic perturbations, such as forest exploitation and extensive livestock. Meanwhile, Aizkorri and Aralar catchments are protected only at the highlands; thus anthropic pressure intensifies at lower lands, where villages are also found (Appendix S1).

### 2.2 Data collection

Environmental variables, prey availability and fish sampling were spatially replicated (5–10 replicates) in each of the five protected areas from 15 June 2022 to 14 July 2022.

### 2.3 Environmental drivers

Data on riparian forest and habitat complexity were gathered across all protected areas (Appendix S1). Riparian forest quality was described using the Adapted Riparian Quality Index (URA, 2021), and canopy cover percentage was described by means of a Spherical Crown Densiometer (Bain & Stevenson, 1999). Fluvial habitat complexity was assessed following the Stream Heterogeneity Index (IHF, Pardo et al., 2002; Valero et al., 2015) and describing the granulometry (Wolman, 1954). The IHF, with values up to 100, evaluates the habitat heterogeneity and physical variables of the stream channel that are influenced by hydrology and substrate composition (see Pardo et al., 2002 for further details). This index has been widely employed by Spanish water agencies and researchers to evaluate river habitat quality (e.g., Fernández et al., 2011; Valero et al., 2015). For describing granulometry, we randomly collected 100 riverbed substrates in each sampling site and assigned them to the size categories established by Wolman (1954). The median values obtained in each site were used as descriptor of the riverbed granulometry.

### 2.4 Biological drivers

Availability of potential aquatic prey was estimated from five randomly located 0.09 m^2^ Surber samples of benthic invertebrates collected at each site (e.g., Sánchez‐Hernández & Cobo, 2018b; Sánchez‐Hernández et al., 2021a). All invertebrates retained in a 1‐mm sieve were identified to family following Tachet et al. (2010). Benthic macroinvertebrates density (ind./m^2^) and taxa richness (the number of families) were calculated. Moreover, macroinvertebrate densities were combined with the affinity of each family to the locomotion traits from Tachet et al. (2010) to calculate swimmer, crawler and sessile macroinvertebrate densities.

Fishes were collected using pulsed D.C. backpack electrofishing equipment (Hans Grassl GmbH, IG200), identified to species level, counted and measured (fork length) to the nearest 1 mm. The density (fish per square meter) of each fish species was roughly calculated using a single‐pass electrofishing to avoid resource‐intensive sampling. Four fish species were captured, including brown trout, Pyrenean stone loach, Pyrenean minnow and European eel (*Anguilla anguilla*). Individuals were returned to the river, except for a subset covering a subsample representative of a length‐frequency distribution of each population, which were immediately killed using cerebral concussion followed by an overdose of anaesthetic (protocols were approved by the Animal Care Committee of the University of the Basque Country‐M20/2022/136). Fish individuals were stored at −20°C for later laboratory analysis of their feeding habits. Although the European eel was captured at several sampling sites, individuals were only euthanised in one river system (Aralar), and it was dropped from subsequent analyses. Pyrenean stone loach was only captured in three out of five protected areas. In total, 345 individuals were used for feeding analyses. Because fish populations are often regulated by density‐dependent mechanisms (Grossman & Simon, 2020; Henderson & Magurran, 2014), fish density – both within and among species – can serve as an indicator of intra‐ and interspecific competition, respectively (e.g., Hasegawa, 2016; Sánchez‐Hernández et al., 2021a). To capture this, we considered total fish density within communities and further partitioned it into species‐specific densities for brown trout, Pyrenean stone loach and Pyrenean minnow as proxies for intra‐ and interspecific competition.

### 2.5 Feeding analyses

The 345 guts were opened to first visually determine the total fullness, ranging from empty (0%) to full (100%) according to the relative fullness method (Amundsen & Sánchez‐Hernández, 2019), and to assign then relative contribution of each food resource to the total stomach fullness. Food resources were classified as aquatic prey, surface prey (i.e., terrestrial arthropods and emerged aquatic insects), non‐animal (detritus and vegetal rest) and fish remains (scales). Thus, prey categories were also used to inform about foraging modes (benthic and surface‐drift foragers), assuming that benthic foraging included individuals feeding mainly on aquatic prey, whereas individuals performing surface‐drift foraging feed chiefly surface prey (e.g., Sánchez‐Hernández et al., 2016; Sánchez‐Hernández & Cobo, 2018a, 2018b). Animal prey items in each gut were identified to the same taxonomic level (mostly family, Appendix S2) as prey availability.

Niche width at the population level was calculated using Levins' index. The proportional similarity index (PSi) was calculated to study the individual dietary specialisation (Bolnick et al., 2002). This index compares each individual's diet with the diet at the population level, with values ranging between 0 and 1. For individuals specialising on a single or few prey items, the PSi values tend to be low, whereas for individuals that consume resources in a similar proportion as the entire population, the PSi values approach 1 (Bolnick et al., 2002).

### 2.6 Statistics

All data analyses were conducted in R version 4.3.1 (R Core Team, 2023). Dietary niche variation at the population (Levins’) and individual (PSi) levels was calculated using the *RinSp* package (Zaccarelli et al., 2013). Graphical visualisations were conducted using the *ggplot2* package version 3.5.1 (Wickham, 2016) and *rstatix* package version 0.7.2 (Kassambara, 2023) to perform Tukey's post hoc tests related to fish feeding and individual niche variation (hypothesis 1).

We employed multiple regression modelling using generalised linear models (GLMs) with quasi‐binomial distribution and the default logit link function to examine the effects and identify the most influential environmental and biological drivers shaping feeding patterns (hypothesis 2) – specifically, the proportions of aquatic and surface prey – and individual niche variation (hypothesis 3) across the three fish species. In total, we fitted nine models (three response variables × three species), using trophic niche components – proportion of aquatic prey, proportion of surface prey and PSi – as response variables. Explanatory variables included environmental factors (canopy cover, substrate granulometry and IHF) and biological factors (benthic macroinvertebrate richness, total macroinvertebrate density, crawler, swimmer and sessile macroinvertebrate densities, total fish density, species‐specific fish densities and fish length).

Prior to modelling, variance inflation factors (VIF) were calculated to assess multicollinearity, that is, correlations among predictor variables. Specifically, we evaluated collinearity among (i) IHF and other environmental drivers (canopy cover and granulometry), (ii) total macroinvertebrate density and the partial densities associated with locomotion traits (crawler, swimmer and sessile macroinvertebrates) and (iii) total fish density and species‐specific fish densities (brown trout, Pyrenean stone loach and Pyrenean minnow densities) using the *car* package (Fox & Weisberg, 2019). In most cases, we found VIF values smaller than 3, indicating low evidence for collinearity (Zuur et al., 2010). Accordingly, the densities of Pyrenean stone loach and Pyrenean minnow were not included in the GLMs (Appendix S3). Thus, to explore the effects of the drivers on feeding (PSi and proportion of both aquatic and surface prey) of each fish species, we first built a full model, including three environmental drivers (granulometry, canopy cover and IHF) and eight biological drivers (benthic macroinvertebrate richness, total macroinvertebrate density, crawler, swimmer and sessile macroinvertebrate densities, total fish density, brown trout density and fish size) as explanatory variables. Next, we selected the best model simulations for each response variable (aquatic prey, surface prey and PSi) using the *backwd_stepwise_glm()* function (*AutoStepwiseGLM* package version 0.2.0; England, 2018) based on the full model. The simplified models were ranked based on their Akaike information criterion (AIC), which seeks the model based both on its goodness of fit and its simplicity, with the model with the lowest AIC value considered the best trade‐off. We visually inspected residuals of the final selected models for deviations from normality and heteroscedasticity (Appendix S4). The configuration of best model simulations, along with the variance explained by each driver of those selected models, allowed us to assess whether the drivers influence all species equally. A significance level of *p* = 0.05 was used for all analyses.

## 3 RESULTS

### 3.1 Feeding and niche variation

**FIGURE 2:**
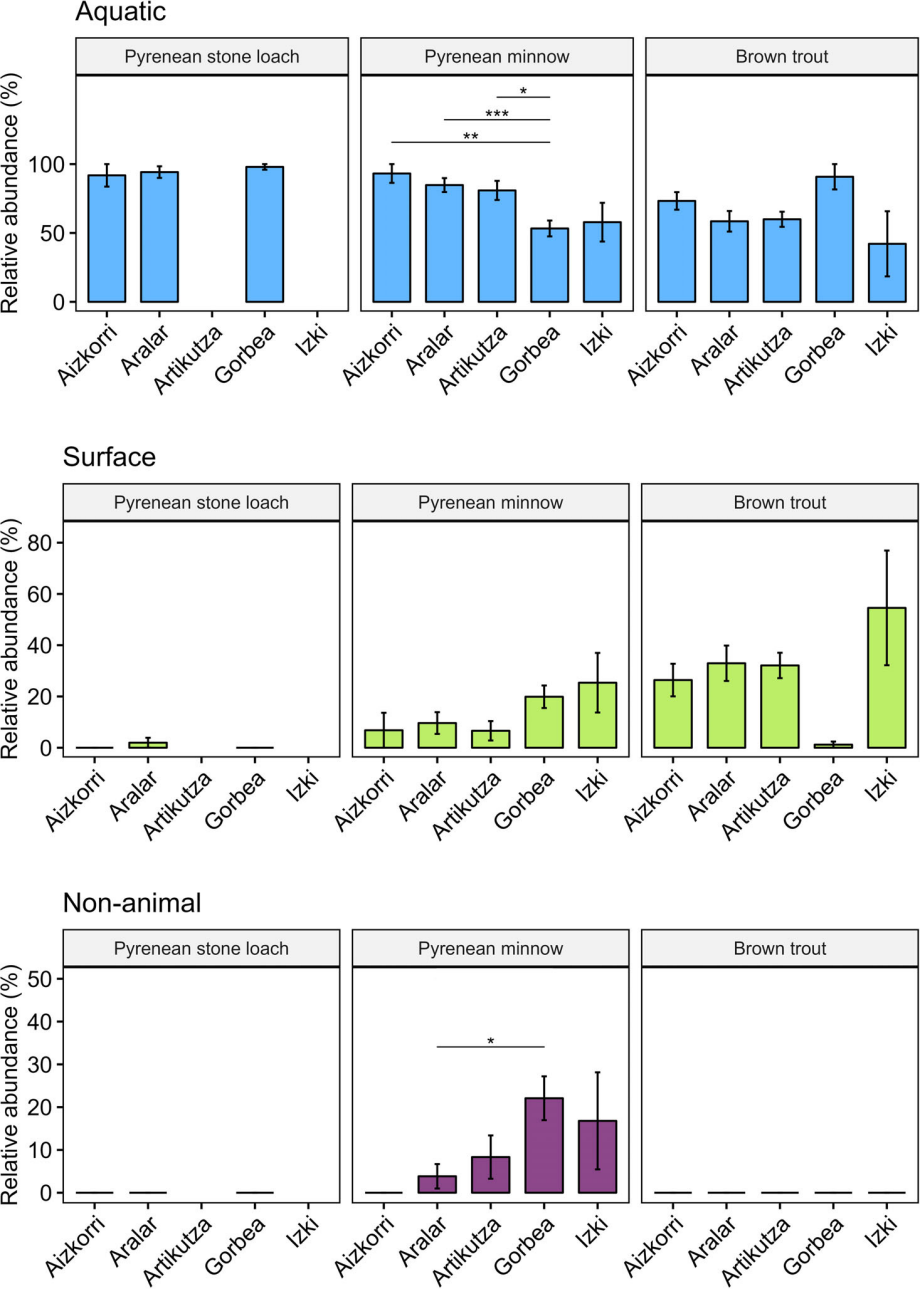
Estimated means for the proportion of different prey categories in the gut contents for brown trout (*Salmo trutta*), Pyrenean minnow (*Phoxinus bigerri*) and Pyrenean stone loach (*Barbatula quignardi*). Error bars represent 95% confidence intervals. Only statistically significant pair‐wise comparisons are reported (*p < 0.05; **p < 0.01; ***p < 0.001).

**FIGURE 3:**
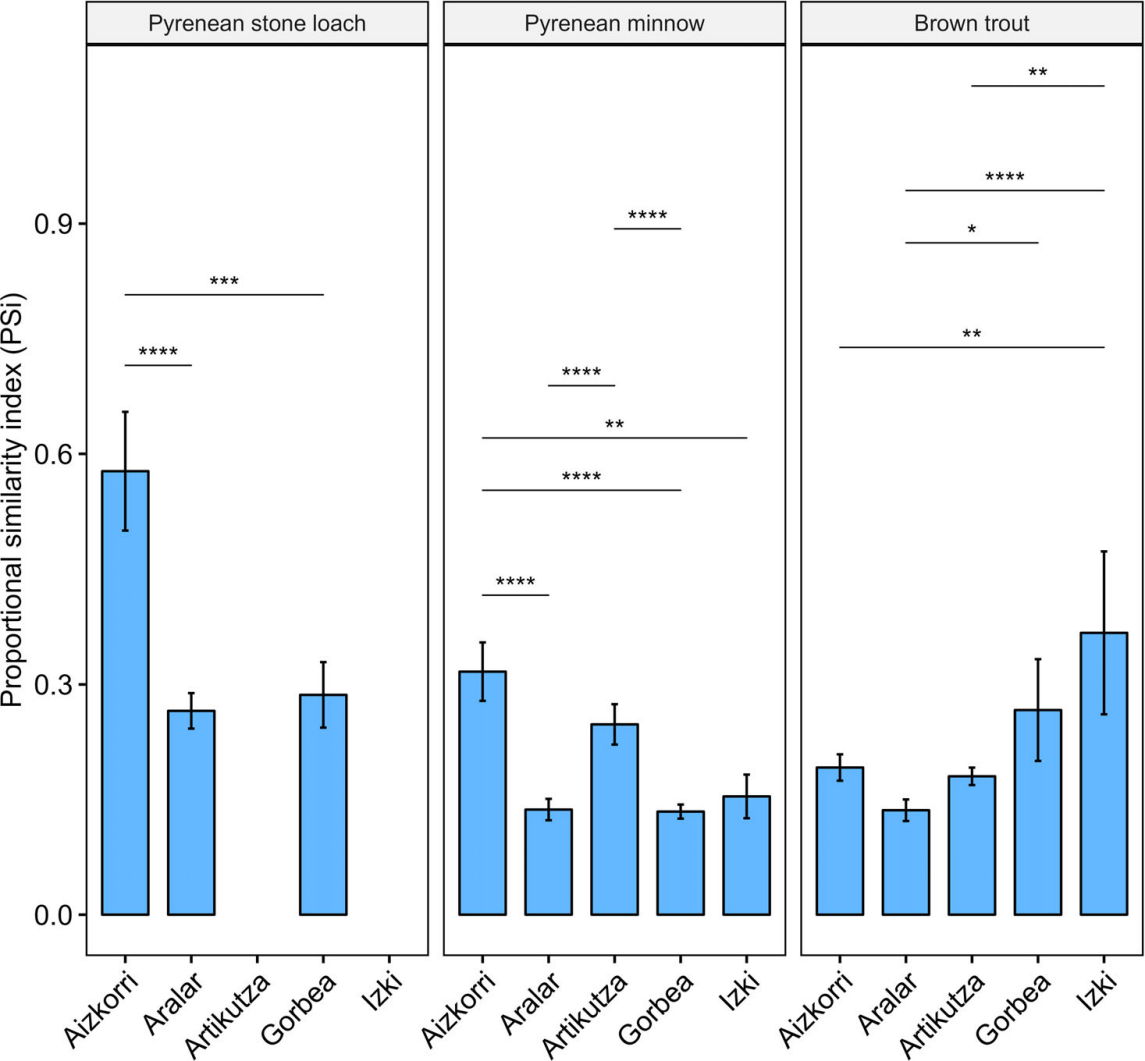
Estimated means for individual diet specialisation quantified as the proportional similarity index (PSi) of the diet utilisation for brown trout (*Salmo trutta*), Pyrenean minnow (*Phoxinus bigerri*) and Pyrenean stone loach (*Barbatula quignardi*). Error bars represent 95% confidence intervals. Low PSi values indicate a high level of individual specialisation. Only statistically significant pair‐wise comparisons are reported (*p < 0.05; **p < 0.01; ***p < 0.001; ****p < 0.0001).

We observed distinct patterns in feeding and niche variation among the fish species (Figure 2). Pyrenean stone loach fed almost exclusively on aquatic prey showing a clear benthic foraging, whereas Pyrenean minnow was the sole species consuming non‐animal resources in combination with both aquatic and surface prey (Figure 2). Except in one protected area (Gorbea), brown trout showed the highest reliance on terrestrial invertebrates (Figure 2). The trophic niche width varied from 20.5 for brown trout in Artikutza to 3.1 for Pyrenean stone loach in Aizkorri (Appendix S5). In general, the niche width of Pyrenean stone loach was considerably lower than those of the other species, whereas this index was higher in Pyrenean minnow than in brown trout (except in Aizkorri and Artikutza; Appendix S5). Regarding the PSi, Pyrenean stone loach had the highest PSi values (i.e., the lowest degree of individual specialisation), whereas brown trout and Pyrenean minnow were more specialised (Figure 3). Brown trout in Aizkorri and Artikutza generally had lower PSi values than Pyrenean minnow, but Pyrenean minnow revealed lower PSi values than brown trout in Gorbea and Izki (Figure 3 and Appendix S6).

### 3.2 Species‐specific drivers explaining feeding

**FIGURE 4:**
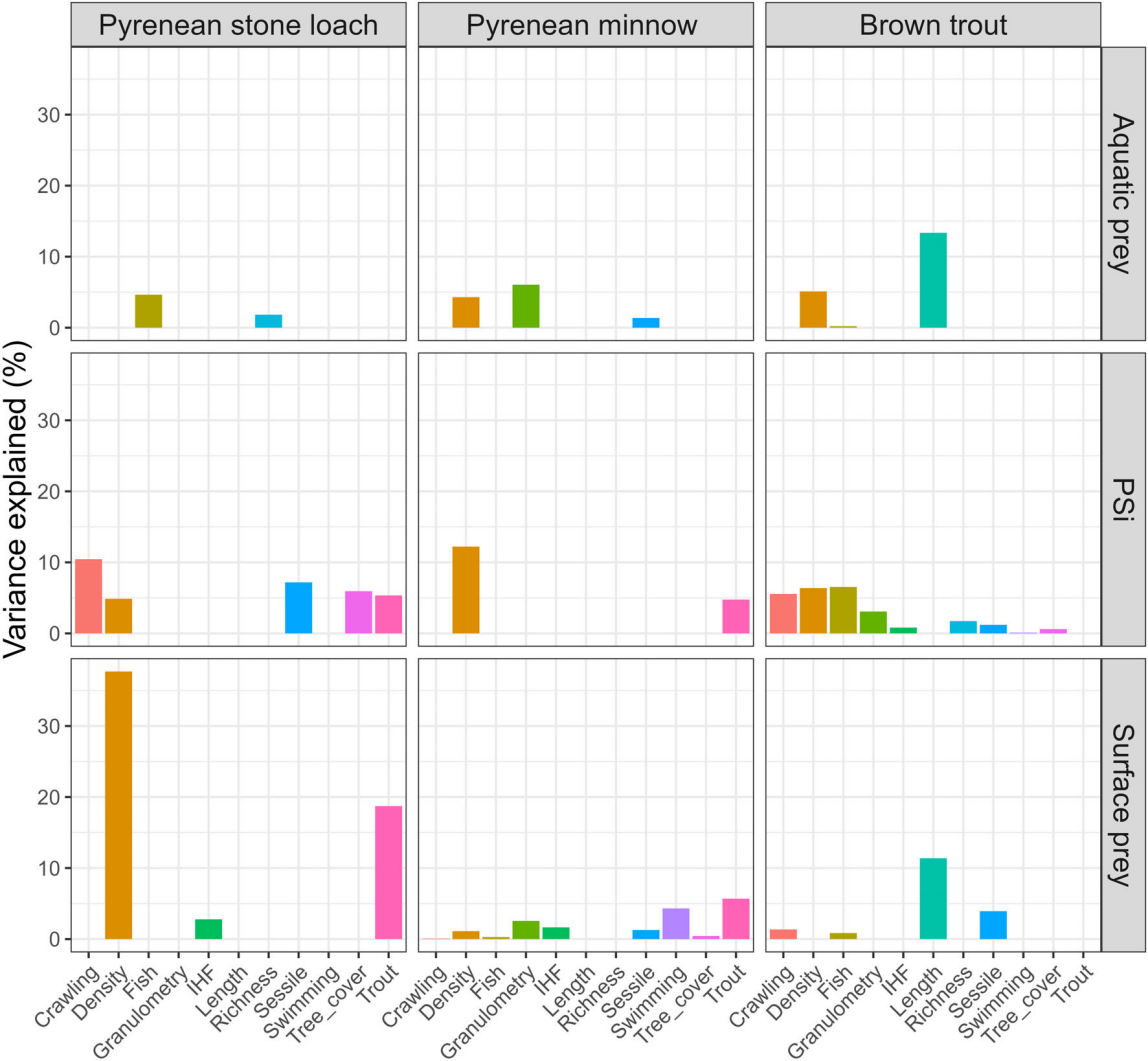
Variance explained by each driver for brown trout (*Salmo trutta*), Pyrenean minnow (*Phoxinus bigerri*) and Pyrenean stone loach (*Barbatula quignardi*) with respect to aquatic prey, surface prey and the proportional similarity index (PSi). Crawling = density of crawling macroinvertebrates; Density = macroinvertebrate density; Fish = fish density; Granulometry = substratum roughness; IHF = Stream Heterogeneity Index; Length = fork length; Richness = benthic macroinvertebrate richness; Sessile = density of sessile macroinvertebrates; Swimming = density of swimming macroinvertebrates; Tree_cover = canopy cover; Trout = brown trout density.

**FIGURE 5:**
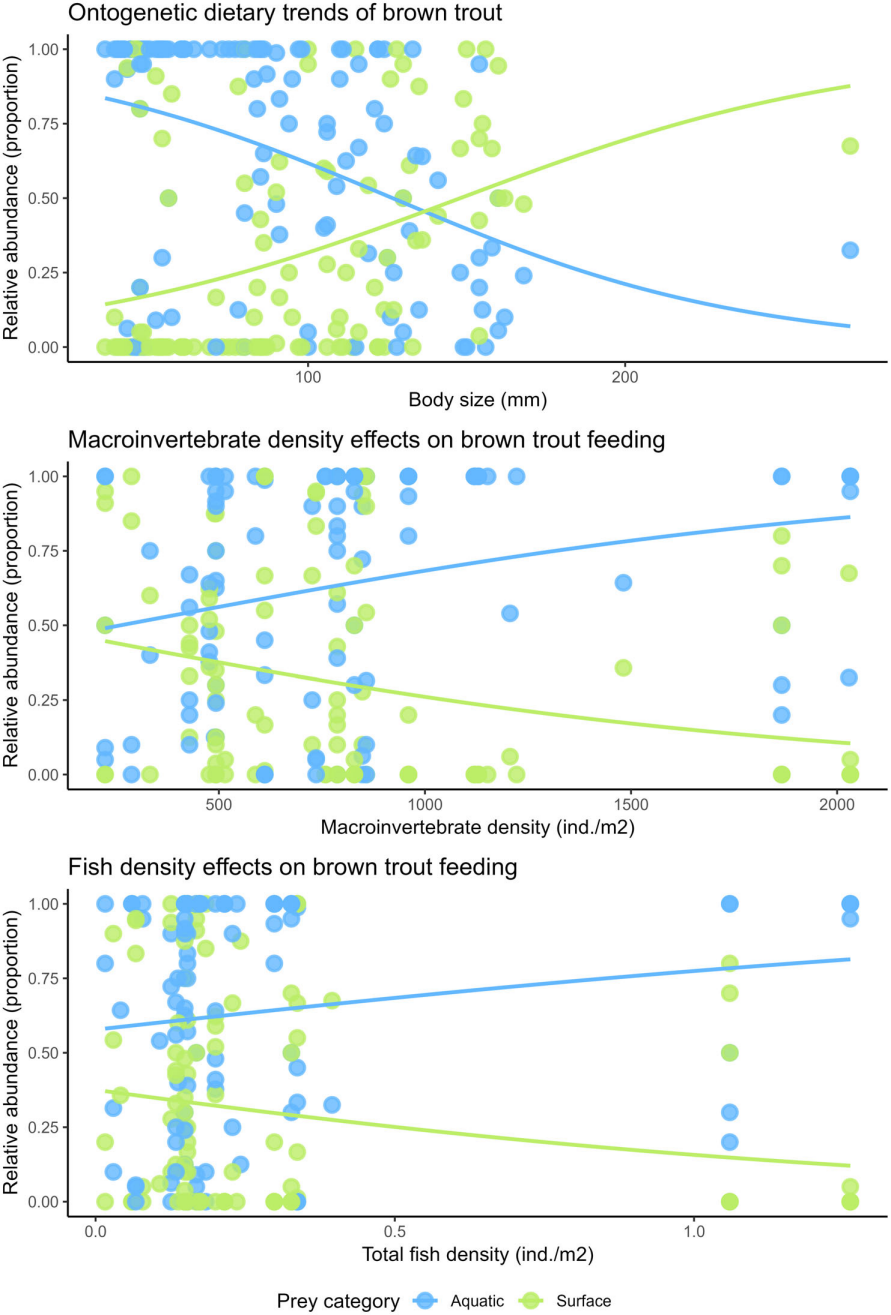
Generalised linear models (GLMs) with quasi‐binomial distribution explaining the association between feeding (aquatic and surface prey) of brown trout and its significant drivers. Note panels only included the statistically significant predictors based on Table 1.

Benthic macroinvertebrate density was the most frequent driver of fish feeding, appearing in seven out of nine best model configurations (Table 1). However, the identity of the main drivers of fish feeding depended on the analysed fish species and prey categories (Figure 4). For example, the variable with the highest power to explain variations in aquatic feeding of Pyrenean minnow was granulometry, whereas it was fish length in brown trout (Figure 4). There was an influence of fish length, benthic macroinvertebrate density and total fish density on the feeding of brown trout (Table 1), with effects on aquatic feeding clearly different from those on surface prey feeding (Figure 5). The consumption of aquatic prey by Pyrenean minnow was positively associated with benthic macroinvertebrate density but negatively with sessile macroinvertebrate density and granulometry, whereas the consumption of surface prey was negatively associated with brown trout density (Table 1). In contrast, no significant effects of either environmental or biological drivers on the feeding of Pyrenean stone loach were found (Table 1).

**TABLE 1:** Summary table of the best model simulations for the proportional similarity index (PSi) and proportions of aquatic and surface prey for each fish species according to the model selection method (automated backward stepwise).

	Variable	Intercept	Environmental drivers			Biological drivers							
			Granulometry	Canopy cover	IHF	Benthic macroinvertebrate richness	Benthic macroinvertebrate density	Benthic macroinvertebrate density (crawling)	Benthic macroinvertebrate density (sessile)	Benthic macroinvertebrate density (swimming)	Total fish density	Brown trout density	Fish length
ST	Aquatic	1.399*	—	—	—	—	0.002**	—	—	—	−1.723*	—	−0.018***
	Surface	−1.012	—	—	—	—	—	−0.004	−0.011*	—	0.411	—	0.014**
	PSi	−0.205	−0.009***	−0.009	0.012	−0.059*	0.002***	−0.004	0.001	−0.006	−1.077**	—	—
PSL	Aquatic	4.803	—	—	—	−0.033	—	—	—	—	−1.365	—	—
	Surface	24.900	—	—	−1.206	—	0.031	—	—	—	—	−11.180	—
	PSi	1.890	—	−0.022	—	—	−0.004***	0.0184**	0.005*	—	—	13.232***	
PM	Aquatic	1.343**	−0.009**	—	—	—	0.001*	—	−0.005*	—	—	—	—
	Surface	−1.602	−0.011	0.039	0.0271	—	−0.001	0.009	0.001	−0.154	1.361	−18.860**	0.002
	PSi	−1.094***	—	—	—	—	−0.001***	—	—	—	—	3.289**	—

*Note*: The parametric coefficients with significance values are given for each variable.

Abbreviations: IHF, Stream Heterogeneity Index; PM, Pyrenean minnow (*Phoxinus bigerri*); PSi, the proportional similarity index; PSL, Pyrenean stone loach (*Barbatula quignardi*).

* *p* < 0.05.

** *p* < 0.01.

*** *p* < 0.001.

### 3.3 Species‐specific drivers explaining individual niche variation

Best model configurations for PSi recognised the importance of environmental and biological drivers for explaining individual specialisation in feeding, but drivers were species specific (Table 1; Figure 4). For example, benthic macroinvertebrate density had a positive influence on PSi of brown trout but a negative influence on Pyrenean minnow and Pyrenean stone loach, whereas total fish density had only a negative influence on brown trout. In contrast, brown trout density had a positive influence on PSi of Pyrenean minnow and Pyrenean stone loach (Table 1).

## 4 DISCUSSION

Brown trout and Pyrenean stone loach had narrower trophic niches compared to the Pyrenean minnow, which displays higher niche width and specialisation variability across the protected areas. These differences can be explained by their inherent foraging strategies: brown trout normally acts as specialised predator, Pyrenean stone loach is benthivore and Pyrenean minnow is omnivorous (Doadrio, 2001; Oscoz et al., 2001, 2004; Sánchez‐Hernández & Cobo, 2018b). Nevertheless, our multiple regression analyses revealed that environmental and biological factors bend general fish feeding and individual specialisation patterns. Moreover, these drivers appear to be species specific, as we did not observe that these factors influenced the feeding patterns of the Pyrenean stone loach, whereas we observed that they had clearer effects on larger and more mobile fish species such as brown trout and Pyrenean minnow.

### 4.1 Feeding and niche variation

Our first hypothesis predicting that fish species exhibit distinct feeding strategies leading to differences in niche variation was supported by the results. Consistent with previous studies, we found that brown trout predominantly consumed surface prey (Sánchez‐Hernández & Cobo, 2018a, 2018b), Pyrenean stone loach primarily fed on aquatic macroinvertebrates (Oscoz et al., 2004), and Pyrenean minnow exhibited an omnivorous diet, incorporating both animal and non‐animal resources (Oscoz et al., 2001, 2008). It should be kept in mind that the Pyrenean stone loach is benthivore and primarily occupies microhabitats such as sheltered substrate areas for feeding and avoiding predators (Prenda et al., 1997), suggesting that its feeding behaviours and ecological interactions are highly localised and specific to certain types of habitats. Thus, these results are in accordance with the proposal made by Worischka et al. (2015) that diet in benthivore fish species is better linked to benthic macroinvertebrate metrics (macroinvertebrate size, abundance and locomotion mode) than to habitat complexity (microhabitat and current velocity preference). It is possible that this kind of species (small, benthivore and little mobile), such as loaches (e.g., *Cobitis* spp. and *Barbatula* spp.) and other small fish (e.g., *Cottus* spp. and *Gobio* spp.), requires approaches at micro‐ and meso‐habitat scales to disentangle the role of environmental and biological drivers in its feeding (Copp & Vilizzi, 2004; MacKenzie & Greenberg, 1998; Wegscheider et al., 2020), scale resolutions that have not been considered in the design of the current study.

### 4.2 Species‐specific drivers explaining feeding

Our second hypothesis was also supported, as species‐specific drivers accounted for the observed dietary differences among the three fish species. Our models reveal the positive effect of benthic macroinvertebrate density on feeding on aquatic prey by both brown trout and Pyrenean minnow. This outcome is supported by previous studies showing the link between prey availability and fish diet (Kreiling et al., 2021; Sánchez‐Hernández & Cobo, 2018a): when aquatic macroinvertebrates are more abundant and accessible than surface prey, brown trout individuals do not specialise on surface prey (surface‐drift foraging) and feed mainly on aquatic prey. In addition, we observed that the consumption of aquatic prey by Pyrenean minnow was negatively associated with density of sessile prey, demonstrating that sessile macroinvertebrates seemed to be avoided by this omnivorous fish species. We posit that its feeding depends on the ability to detect either sessile macroinvertebrates or more preferred food resources when available.

We observed a significant effect of total fish density only on the feeding of brown trout: as total fish density increased, aquatic feeding increased and terrestrial feeding decreased. Theory predicts that fish densities (as a proxy of inter‐ and intraspecific competition) have an impact on feeding and niche variation in stream‐dwelling salmonids (Boujard et al., 2002; Karjalainen, 1992; Sánchez‐Hernández & Cobo, 2018b). For example, Sánchez‐Hernández and Cobo (2018b) observed that increasing brown trout density was associated with a reduced proportion of surface prey in their diet. Notably, although most salmonid species have shown evidence of density dependence in growth, mortality, fecundity and recruitment (Grossman & Simon, 2020), our findings indicate that such effects may not uniformly extend to feeding dynamics in this context. That is, we found no significant effect of brown trout density – used as a proxy for intraspecific competition – on feeding behaviour; but total fish density had a negative influence on surface‐drift foraging. This suggests that interspecific competition might play a more prominent role than intraspecific interactions in shaping brown trout feeding strategies, particularly in their preference between benthic and surface‐drift prey as indicated by the findings of the present study.

Only brown trout showed an influence of fish length on the feeding. Salmonids commonly exhibit size‐structured dominance hierarchies, where larger individuals dominate smaller ones, leading to differences in feeding behaviour between dominant and subordinate individuals (e.g., Kohda et al., 2008; Nakano, 1995; Nakano et al., 1999). The observed pattern, that is, the increasing reliance of allochthonous resources (terrestrial invertebrates) with ontogeny, matches with previous studies focusing on brown trout (Sánchez‐Hernández & Cobo, 2016) and on stream‐dwelling salmonids in general (reviewed by Sánchez‐Hernández, 2024). In contrast, the lack of ontogenetic effect on the feeding of Pyrenean stone loach and Pyrenean minnow may be attributable to morphological constrains linked to mouth gape limitation, which prevents the consumption of alternative and bigger prey types, and the limitations when capturing large individuals. A fish's ability to handle prey generally scales with mouth gape size, which in turn typically scales with its body size (Sánchez‐Hernández, Nunn, et al., 2019). Specifically, Pyrenean stone loach, a small benthivore species, likely does not change diet composition through ontogeny but changes occur depending on prey size. Although we can attribute the lack of any ontogeny shift to the fact that we did not observe the largest individuals recorded for this species (120 mm of maximum body length; Leunda et al., 2017), Oscoz et al. (2006) also observed a similar diet composition of this species irrespective of body size.

### 4.3 Species‐specific drivers explaining individual niche variation

Our third hypothesis predicting that drivers of individual specialisation are species specific was also supported; as, for example, our models found that total fish density had a negative influence on PSi in brown trout, but brown trout density had a positive influence on PSi of Pyrenean stone loach and Pyrenean minnow. This supports the view that resource competition promotes niche variation among individuals within a population (e.g., Araújo et al., 2011; Svanbäck & Bolnick, 2007; Costa‐Pereira et al., 2019; Sánchez‐Hernández et al., 2021a). More specifically, species‐specific differences found in the current study may be linked to the differences in the inherent feeding habits of each fish species, with less‐important effects of fish density on niche variation in omnivorous fish species (as it is the case for the Pyrenean minnow) compared to those species relaying exclusively on animal resources.

We also found that PSi of brown trout increased with macroinvertebrate density but decreased with macroinvertebrate richness. This finding partially supports previous research on Scandinavian brown trout populations (Sánchez‐Hernández et al., 2021a), which reported a negative influence of benthic macroinvertebrate richness on PSi, although no statistically significant relationship was observed between benthic macroinvertebrate density and PSi. Behavioural diversification in feeding is generally driven by prey diversity under scenarios with no extensive food resource limitation, whereas prey density may become more important when resource limitations are severe (Sánchez‐Hernández et al., 2021a). It is possible that reported macroinvertebrate densities in the current study (224–2032 ind./m^2^, including only macroinvertebrates higher than 1 mm) are low compared to other more productive in similar rivers of northern Iberian Peninsula (Barba et al., 2010), which can lead to individual niche diversification (Araújo et al., 2011; Sánchez‐Hernández et al., 2021a).

In brown trout, rougher substrate was linked to a higher individual specialisation. This can be linked to higher substratum roughness reducing the conspicuousness and accessibility of aquatic invertebrates to brown trout (De Crespin Billy & Usseglio‐Polatera, 2002), making individuals more specialised in foraging and feeding. In contrast, higher substratum roughness is linked to higher consumption of aquatic prey in Pyrenean minnow, likely as a consequence of its higher reliance on prey attached to the substratum such as Ancylidae (see Appendix S2).

## 5 CONCLUSIONS

Our study provides a novel way to model the links between environmental and biological drivers and fish feeding in riverine systems, showing species‐specific effects to explain differences in diet and individual niche variation in fish. Among all the environmental factors, granulometry was the main driver explaining variations in feeding and individual niche differences in all three species. Among biological factors, benthic macroinvertebrate density emerged as the most frequent driver of fish feeding. Thus, fish conservation and management plans should not only focus on direct actions as stocking but also on restoration works to increase habitat quality to stimulate production of benthic macroinvertebrates. These actions will lead long‐term ecological benefits that directly support fish populations by balancing prey availability and resource competition. The ways in which different fish species adapt their feeding strategies to various environmental and ecological drivers underscore the importance of expanding research beyond predatory species, particularly on omnivorous and endemic species. In this regard, the limited published literature on the trophic ecology of species other than brown trout (i.e., Pyrenean minnow and Pyrenean stone loach) has constrained our ability to discuss these species in equal depth. This highlights the need for greater attention to less‐studied species, which, despite their lower socio‐economic importance, play crucial ecological roles within fish assemblages. Generating restoration actions that target multiple fish species represents a challenge for the next generation of fish ecologists and managers.

## AUTHOR CONTRIBUTIONS

Javier Sánchez‐Hernández performed the data analysis and wrote the first draft of the manuscript. All authors commented on previous versions, read and approved the final manuscript.

## FUNDING INFORMATION

This work was supported by the Spanish Ministry of Science, Innovation and Universities through the project PID2020‐115830GB‐100 (RiMSEC) and the Basque Government (Consolidated Research Group IT1471‐22). The authors also acknowledge the Spanish Ministry of Science, Innovation and Universities (I de Guzmán, PID2020‐115830GB‐100 and TED2021‐129966B‐C31), as well as financial support from pre‐doctoral (I Fernandez de Larrea, PIF21/172) and post‐doctoral fellowships (I de Guzman, POSTUPV24/47) from the University of the Basque Country.

## CONFLICT OF INTEREST STATEMENT

The authors declare no conflict of interest.

## Supporting information


**Appendix S1.** Supporting information.

## Data Availability

Data will be made available on reasonable request.
