# Supplementary material for: Species‐specific drivers explain fish feeding and individual niche variation
Source: J Fish Biol. 2025 Jul 13;107(4):1420–32. doi: 10.1111/jfb.70136 (PMC12536044; doi:10.1111/jfb.70136)
Supplement: Supplementary file 1 — Appendix S1. Supporting information. [file JFB-107-1420-s001.docx]

**Supplementary information to “Species-specific drivers explain fish feeding and individual niche variation”**

Javier Sánchez-Hernández, Iñaki Fernández de Larrea, Ioar de Guzmán, José M. González & Aitor Larrañaga

**List of items in the supplementary:**

**-Appendix 1.** Environmental and biological characteristics of studied sites.

**-Appendix 2.** Diet composition for each fish species.

**-Appendix 3.** Multicollinearity: variance inflation factors (VIF).

**-Appendix 4.** Residuals of the best model simulations for the proportional similarity index (PSi) and proportions of aquatic and terrestrial prey for each fish species.

**-Appendix 5.** Dietary niche variation at the population and individual levels for each fish species.

**-Appendix 6.** Pairwise comparisons between species.

**Appendix 1.** Environmental and biological characteristics of studied sites.

**Table S1.** Main characteristics of studied sites and measured environmental and biological variables. Riparian forest quality was described using the Adapted Riparian Quality Index (RQIA) and the fluvial habitat complexity was assessed following the Stream Heterogeneity Index (IHF). Mean ± standard error.

|  |  | **Aizkorri** | **Aralar** | **Artikutza** | **Gorbea** | **Izki** |
| --- | --- | --- | --- | --- | --- | --- |
| Surface (Km^2^) |  | 53.3 | 56.69 | 45.13 | 39.17 | 43.58 |
| Proportion of main land uses | Natural forest | 0.44 | 0.6 | 0.69 | 0.38 | 0.8 |
|  | Plantations | 0.26 | 0.18 | 0.15 | 0.21 | 0.07 |
|  | Grassland | 0.16 | 0.12 | 0.04 | 0.11 | 0.01 |
|  | Shrubland | 0.12 | 0.07 | 0.13 | 0.28 | 0.08 |
| Environmental variables | IHF | 72.56 ± 2.57 | 70.5 ± 1.88 | 73.7 ± 2.59 | 67.8 ± 1.44 | 60 ± 1.24 |
|  | RQIA | 111.89 ± 4.92 | 101.3 ± 5.89 | 118 ± 2.66 | 105.1 ± 3.23 | 122.6 ± 1.77 |
|  | Canopy cover (%) | 76.94 ± 2.06 | 78.12 ± 3.53 | 78.61 ± 3.22 | 68.06 ± 4.35 | 79.96 ± 1.49 |
|  | Granulometry | 83.44 ± 7.44 | 117.33 ± 15.5 | 75.7 ± 4.09 | 173.2 ± 37.85 | 10.24 ± 3.57 |
| Biological variables | Benthic macroinvertebrate density (ind./m^2^) | 442.22 ± 65.32 | 885.78 ± 125.62 | 465.11 ± 74.42 | 876 ± 66.59 | 471.11 ± 46.01 |
|  | Benthic macroinvertebrate richness | 24.0 ± 1.64 | 27.3 ± 1.54 | 21.4 ± 1.3 | 27.1 ± 1.2 | 18.8 ± 1.78 |
|  | Total fish density (ind./m^2^) | 0.16 ± 0.03 | 0.48 ± 0.14 | 0.13 ± 0.03 | 0.14 ± 0.02 | 0.17 ± 0.03 |
|  | Fish length (mm) | 73.3 ± 24.42 | 91.6 ± 54.15 | 90.6 ± 35.99 | 68.7 ± 18.67 | 90.4 ± 31.41 |

**Appendix 2.** Diet composition for each species.

**Table S2.** Diet composition (relative abundance, %). BT = brown trout (*Salmo trutta*), BQ = Pyrenean stone loach (*Barbatula quignardi*) and PB = Pyrenean minnow (*Phoxinus bigerri*).

| **River** | **Aizkorri** | | |  | **Aralar** | | |  | **Artikutza** | |  | **Gorbea** | | |  | **Izki** | |
| --- | --- | --- | --- | --- | --- | --- | --- | --- | --- | --- | --- | --- | --- | --- | --- | --- | --- |
| **Taxa** | BQ | PB | ST |  | BQ | PB | ST |  | PB | ST |  | BQ | PB | ST |  | PB | ST |
| **Aquatic prey** |  |  |  |  |  |  |  |  |  |  |  |  |  |  |  |  |  |
| Baetidae | 12.9 | 18.6 | 25.9 |  | 11.0 | 17.3 | 18.8 |  | 22.3 | 2.6 |  | 7.8 | 4.6 | 1.8 |  | 9.2 | 1.4 |
| Heptageniidae | 3.0 | - | 8.0 |  | 16.6 | 6.9 | 13.7 |  | 3.4 | 8.2 |  | 11.5 | 2.0 | 8.9 |  | - | 2.4 |
| Ephemerellidae | - | - | - |  | 0.1 | - | 0.3 |  | - | 0.4 |  | 3.8 | - | 24.4 |  | - | - |
| Ephemeridae | - | - | - |  | 0.3 | - | - |  | - | - |  | - | 3.5 | - |  | 17.4 | - |
| Ephemeroptera | 4.0 | - | 1.0 |  | 0.6 | 0.8 | 2.9 |  | 0.9 | 0.2 |  | 8.2 | 1.5 | - |  | 1.1 | 1.1 |
| Perlidae | - | - | - |  | - | - | - |  | - | 3.1 |  | - | - | 44.4 |  | - | - |
| Neumoridae | - | - | - |  | 1.0 | - | - |  | - | - |  | - | - | - |  | - | - |
| Leuctridae | - | - | 3.6 |  | 1.0 | - | - |  | - | 0.2 |  | 1.6 | - | - |  | - | - |
| Plecoptera | - | - | 0.5 |  | 0.8 | - | - |  | 2.1 | 0.3 |  | 3.6 | 3.7 | - |  | - | - |
| Philopotamidae | 3.0 | - | 1.1 |  | 2.5 | 3.6 | - |  | 4.3 | 2.2 |  | - | 1.9 | - |  | 8.6 | - |
| Polycentropodidae | - | - | 2.3 |  | - | 0.6 | 2.4 |  | - | - |  | 1.6 | 0.4 | - |  | - | - |
| Hydropsychidae | 6.9 | 1.4 | 4.2 |  | 4.9 | 9.1 | 2.6 |  | 17.1 | 6.5 |  | 5.6 | 4.2 | - |  | - | - |
| Hydropsychidae (p) | - | - | - |  | - | - | - |  | 4.9 | - |  | - | - | - |  | - | - |
| Rhyacophilidae | 3.0 | - | 0.2 |  | 8.9 | 10.1 | 2.8 |  | 0.6 | 1.1 |  | 13.8 | 5.8 | - |  | - | - |
| Sericostomatidae | - | - | 3.8 |  | - | 0.6 | 1.0 |  | - | 1.7 |  | 1.6 | - | 6.7 |  | - | - |
| Limnephilidae | - | - | - |  | - | 2.5 | 0.5 |  | - | 7.5 |  | - | - | - |  | - | - |
| Hydroptilidae | - | - | - |  | - | 8.9 | 0.2 |  | - | - |  | - | 0.6 | - |  | - | - |
| Leptoceridae | - | - | 0.9 |  | - | - | 0.1 |  | - | 1.0 |  | - | - | - |  | - | - |
| Trichoptera (p) | - | - | - |  | - | - | 1.2 |  | - | - |  | - | - | - |  | - | - |
| Trichoptera | 0.2 | 10.9 | 5.5 |  | 0.6 | 4.4 | 0.2 |  | 0.5 | 2.6 |  | - | 0.2 | - |  | 1.1 | - |
| Chironomidae | 0.8 | 4.1 | 2.8 |  | 16.0 | 1.1 | 0.6 |  | 5.3 | 2.3 |  | 10.7 | 9.7 | 0.4 |  | 3.6 | 1.1 |
| Chironomidae (p) | - | - | 3.1 |  | - | - | 0.5 |  | - | 0.1 |  | - | - | - |  | - | - |
| Blephariceridae | - | - | - |  | - | - | - |  | 0.1 | 0.4 |  | - | - | - |  | - | - |
| Ceratopogonidae | - | - | - |  | - | - | - |  | - | - |  | - | 0.1 | - |  | - | - |
| Empididae | - | - | - |  | - | - | - |  | - | - |  | - | 0.7 | - |  | - | - |
| Limonidae | - | - | - |  | - | - | 0.2 |  | - | 1.1 |  | - | - | - |  | - | - |
| Tipulidae | - | - | - |  | - | - | - |  | - | - |  | - | 1.3 | - |  | - | - |
| Dixidae | - | - | 0.8 |  | - | - | - |  | 1.2 | 0.1 |  | - | - | - |  | - | - |
| Simuliidae | - | 0.7 | 1.6 |  | 7.3 | 1.9 | 1.5 |  | 10.3 | 1.3 |  | 24.2 | 4.3 | - |  | - | 0.3 |
| Simuliidae (p) | 0.4 | - | - |  | - | 1.9 | - |  | - | - |  | 0.5 | - | - |  | - | - |
| Gammaridae | 56.2 | 41.7 | 5.0 |  | 26.1 | 4.5 | 15.6 |  | 4.2 | 10.9 |  | - | 2.0 | - |  | 12.9 | 6.8 |
| Astacidae | - | - | - |  | - | - | - |  | - | - |  | - | 2.3 | - |  | - | 14.1 |
| Elmiidae (a) | - | 2.2 | - |  | - | - | - |  | 0.2 | 1.1 |  | - | 1.0 | - |  | - | - |
| Elmiidae (l) | - | 4.3 | 0.2 |  | - | 0.2 | - |  | 0.9 | 4.1 |  | 0.3 | - | - |  | - | - |
| Dytiscidae (l) | - | - | - |  | - | - | - |  | - | 0.2 |  | - | - | - |  | - | - |
| Hydraenidae (a) | - | - | - |  | - | - | - |  | 1.5 | - |  | - | - | - |  | - | - |
| Coleoptera | - | - | - |  | - | - | - |  | - | - |  | - | 1.5 | - |  | - | - |
| Lymnaeidae | - | - | 0.2 |  | - | - | - |  | - | - |  | - | - | - |  | - | - |
| Ancylidae | - | 3.6 | 0.4 |  | - | - | - |  | 1.8 | 0.6 |  | - | 1.0 | - |  | - | - |
| Hydrobiidae | - | - | 0.6 |  | - | 1.3 | 1.2 |  | - | 4.5 |  | - | - | 3.1 |  | - | - |
| Hydracarina | - | - | - |  | - | 0.9 | - |  | 0.3 | - |  | - | 0.9 | - |  | - | - |
| Unidentified | - | 3.8 | - |  | - | 3.0 | - |  | 3.1 | - |  | 4.2 | 1.3 | - |  | 6.0 | - |
| **Surface prey** |  |  |  |  |  |  |  |  |  |  |  |  |  |  |  |  |  |
| Trichoptera | - | - | 1.1 |  | - | - | 0.2 |  | - | 0.3 |  | - | - | - |  | - | - |
| Tipulidae | - | - | 2.3 |  | - | - | - |  | - | - |  | - | - | - |  | - | - |
| Chironomidae | - | - | 0.6 |  | - | - | - |  | 0.1 | 0.1 |  | - | - | - |  | - | - |
| Empididae | - | - | - |  | - | - | 0.2 |  | - | 0.1 |  | - | - | - |  | 0.9 | - |
| Ephemeroptera | - | - | 0.6 |  | - | - | 0.2 |  | 0.6 | - |  | - | - | - |  | - | - |
| Plecoptera | - | - | - |  | - | - | - |  | - | 0.2 |  | - | - | - |  | - | - |
| Formicidae | - | - | - |  | - | 1.9 | 1.3 |  | - | 4.2 |  | - | 2.5 | - |  | 2.1 | 12.2 |
| Ichneumonidae | - | - | 0.4 |  | - | - | - |  | - | - |  | - | - | - |  | - | - |
| Hymenoptera | - | - | 1.0 |  | - | - | 0.3 |  | 1.4 | 0.5 |  | - | - | - |  | - | - |
| Diptera | - | - | 3.3 |  | - | 3.4 | 2.3 |  | 0.7 | 4.3 |  | - | 6.2 | 1.3 |  | 6.4 | 4.1 |
| Staphylinidae | - | - | - |  | - | - | - |  | - | 0.1 |  | - | - | - |  | - | 1.4 |
| Curculionidae | - | - | - |  | - | - | 2.4 |  | - | 2.0 |  | - | 2.0 | - |  | - | - |
| Coccinellidae | - | - | - |  | - | - | - |  | - | - |  | - | - | - |  | - | - |
| Scarabeidae | - | - | - |  | - | - | - |  | - | 1.5 |  | - | - | - |  | - | - |
| Coleoptera | - | - | 8.4 |  | - | 0.3 | 3.5 |  | - | 5.5 |  | - | 2.1 | - |  | - | 28.4 |
| Cicadellidae | - | - | 0.9 |  | - | - | 0.2 |  | - | 1.5 |  | - | - | - |  | - | - |
| Heteroptera | - | - | 0.8 |  | - | 1.9 | 3.4 |  | - | 0.9 |  | - | - | - |  | - | 14.9 |
| Aphididae | - | - | - |  | - | - | - |  | - | - |  | - | - | - |  | - | - |
| Thysanoptera | - | - | - |  | - | - | - |  | - | 0.1 |  | - | - | - |  | - | - |
| Isopoda | - | - | - |  | - | - | 0.7 |  | - | - |  | - | - | - |  | - | - |
| Myriapoda | - | - | - |  | - | - | 0.7 |  | 2.5 | 2.6 |  | - | - | - |  | 19.3 | - |
| Arachnida | - | 8.7 | 2.5 |  | - | 5.3 | 1.1 |  | - | 2.2 |  | - | 0.7 | - |  | - | - |
| Lepidoptera (l) | - | - | - |  | - | - | 1.9 |  | - | 1.0 |  | - | - | - |  | - | - |
| Pyralidae (l) | - | - | - |  | - | - | - |  | 2.1 | - |  | - | - | - |  | - | - |
| Dermaptera | - | - | - |  | - | - | - |  | - | 0.2 |  | - | - | - |  | - | - |
| Chrysoperla (l) | - | - | - |  | - | - | 0.1 |  | - | - |  | - | - | - |  | - | - |
| Orhoptera | - | - | - |  | - | - | 3.1 |  | - | - |  | - | - | - |  | - | - |
| Unidentified | - | - | 6.1 |  | 2.0 | 2.5 | 7.9 |  | - | 3.7 |  | - | 7.3 | - |  | 0.6 | 8.1 |
| **Other** |  |  |  |  |  |  |  |  |  |  |  |  |  |  |  |  |  |
| Detritus | - | - | - |  | - | - | - |  | 4.9 | - |  | - | 1- | - |  | 2.1 | - |
| Vegetal rests | - | - | - |  | - | 2.5 | - |  | 2.1 | - |  | - | 10.9 | - |  | 8.6 | - |
| Scales | - | - | - |  | - | - | 1.2 |  | - | - |  | - | - | - |  | - | - |
| Unidentified | 9.7 | - | 0.4 |  | 0.3 | 2.6 | 2.6 |  | 0.6 | 4.7 |  | 1.1 | 4.2 | 8.9 |  | - | 4.1 |

**Appendix 3.** Multicollinearity: variance inflation factors (VIF).

**Table S3.** among (i) IHF and other environmental variables (tree cover and granulometry), (ii) total macroinvertebrate density and the partial densities associated with locomotion traits (crawler, swimmer and sessile macroinvertebrates), and (iii) total fish density and species-specific fish densities.

|  | Crawler macroinvertebrate density | Swimmer macroinvertebrate density | Sessile macroinvertebrate density |
| --- | --- | --- | --- |
| Total macroinvertebrate density | 1.06 | 1.04 | 1.03 |
|  | Brown trout density | Pyrenean stone loach density | Pyrenean minnow density |
| Fish density | 1.01 | 4.35 | 4.36 |
|  | Tree cover | Granulometry |  |
| Stream Heterogeneity Index (IHF) | 1.25 | 1.25 |  |

**Appendix 4.** Residuals of the best model simulations for the proportional similarity index (PSi) and proportions of aquatic and terrestrial prey for each fish species.

| Brown trout (*Salmo trutta*) | | |
| --- | --- | --- |
| Aquatic | Surface | PSi |
| 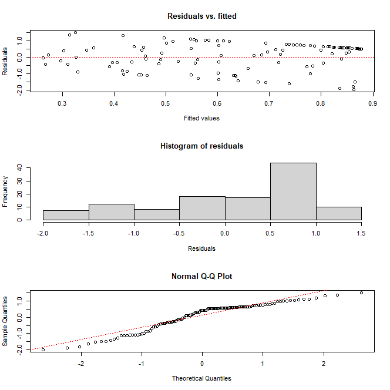 | 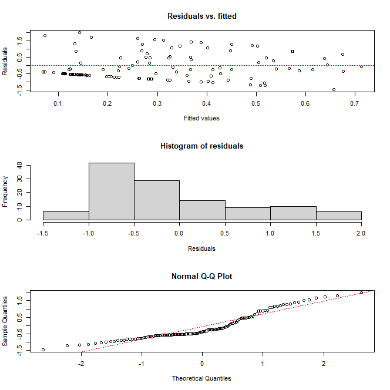 | 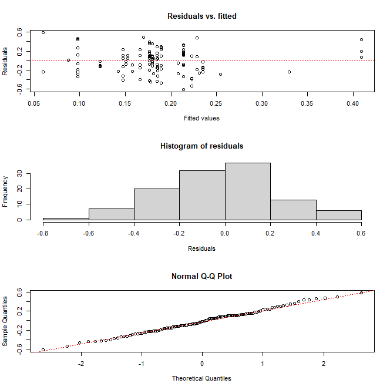 |
| Pyrenean stone loach (*Barbatula quignardi*) | | |
| Aquatic | Surface | PSi |
| 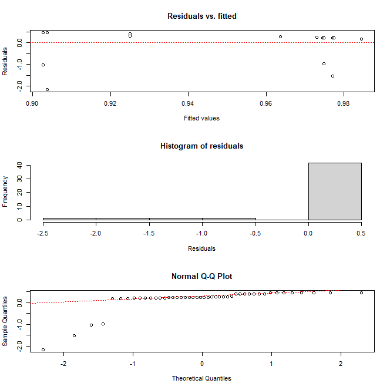 | 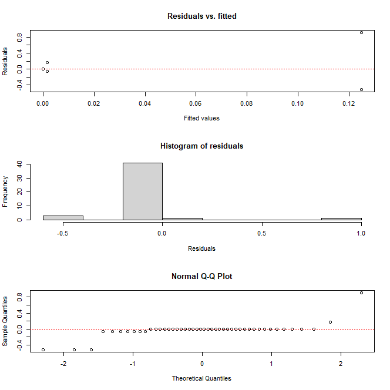 | 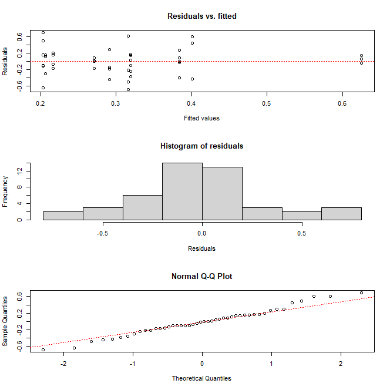 |
| Pyrenean minnow (*Phoxinus bigerri*) | | |
| Aquatic | Surface | PSi |
| 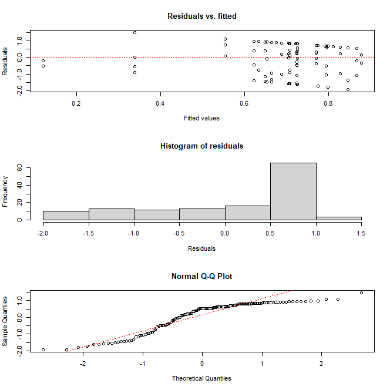 | 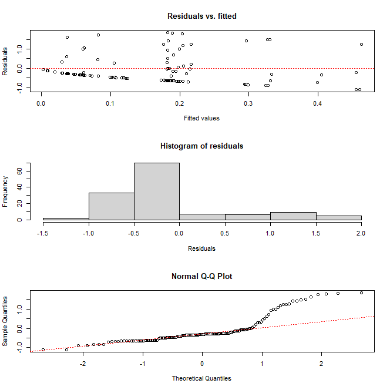 | 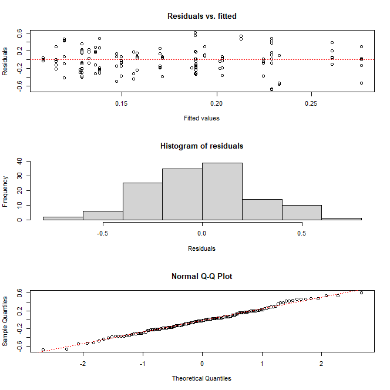 |

**Figure S1.** Residuals of the best model simulations.

**Appendix 5.** Dietary niche variation at the population and individual levels for each fish species.

**Table S4.** Summary of the indices showing dietary niche variation at the population and individual levels of the diet utilisation. Low PSi values indicate high level of individual specialisation.

|  | Sampling size (n) | Proportional similarity index (PSi) | Levins (D) |
| --- | --- | --- | --- |
| **Brown trout**  **(*Salmo trutta*)** |  |  |  |
| Aizkorri | 36 | 0.19 | 10.38 |
| Aralar | 39 | 0.14 | 11.81 |
| Artikutza | 42 | 0.18 | 20.54 |
| Gorbea | 5 | 0.27 | 5.05 |
| Izki | 5 | 0.37 | 7.40 |
| **Pyrenean minnow**  **(*Phoxinus bigerri*)** |  |  |  |
| Aizkorri | 13 | 0.32 | 4.09 |
| Aralar | 51 | 0.14 | 14.40 |
| Artikutza | 24 | 0.25 | 11.20 |
| Gorbea | 67 | 0.13 | 16.92 |
| Izki | 12 | 0.15 | 9.21 |
| **Pyrenean stone loach**  **(*Barbatula quignardi*)** |  |  |  |
| Aizkorri | 6 | 0.58 | 3.15 |
| Aralar | 30 | 0.26 | 6.90 |
| Artikutza | 0 | - | - |
| Gorbea | 15 | 0.29 | 8.25 |
| Izki | 0 | - | - |

**Appendix 6.** Pairwise comparisons between species.

**Table S5**. Pairwise comparisons (Tukey test) between species for the proportional similarity index (PSi) and proportions of food resources (aquatic prey, surface prey and non-animal resources). ST = brown trout (*Salmo trutta*), PB = Pyrenean minnow (*Phoxinus bigerri*) and BQ = Pyrenean stone loach (*Barbatula quignardi*). Significant values are in bold.

|  | group1 | group2 | Aquatic | |  | Surface | |  | Non-animal | |  | PSi | |
| --- | --- | --- | --- | --- | --- | --- | --- | --- | --- | --- | --- | --- | --- |
|  |  |  | Estimate | p-value |  | Estimate | p-value |  | Estimate | p-value |  | Estimate | p-value |
| Aizkorri | BQ | PB | 1.35 | 0.997 |  | 6.82 | 0.913 |  | - | - |  | -0.26 | **<0.001** |
| Aizkorri | BQ | ST | -18.57 | 0.431 |  | 26.40 | 0.176 |  | - | - |  | -0.39 | **<0.001** |
| Aizkorri | PB | ST | -19.92 | 0.211 |  | 19.58 | 0.208 |  | - | - |  | -0.12 | **0.010** |
| Aralar | BQ | PB | -9.42 | 0.528 |  | 7.66 | 0.563 |  | 3.84 | 0.334 |  | -0.13 | **<0.001** |
| Aralar | BQ | ST | -35.71 | **<0.001** |  | 30.98 | **<0.001** |  | 0.00 | 1.000 |  | -0.13 | **<0.001** |
| Aralar | PB | ST | -26.30 | **0.005** |  | 23.32 | **0.003** |  | -3.84 | 0.282 |  | 0.00 | 0.999 |
| Artikutza | PB | ST | -20.95 | 0.021 |  | 25.50 | **<0.001** |  | -8.33 | **0.044** |  | -0.07 | **0.010** |
| Gorbea | BQ | PB | -44.69 | **<0.001** |  | 19.89 | 0.049 |  | 22.08 | 0.063 |  | -0.15 | **<0.001** |
| Gorbea | BQ | ST | -7.16 | 0.925 |  | 1.20 | 0.996 |  | 0.00 | 1.000 |  | -0.02 | 0.919 |
| Gorbea | PB | ST | 37.53 | 0.079 |  | -18.69 | 0.321 |  | -22.08 | 0.309 |  | 0.13 | **0.014** |
| Izki | PB | ST | -15.73 | 0.552 |  | 29.19 | 0.221 |  | -16.80 | 0.300 |  | 0.21 | **0.029** |
